# Subtractive genomics and molecular docking approach to identify drug targets against *Stenotrophomonas maltophilia*

**DOI:** 10.1371/journal.pone.0261111

**Published:** 2021-12-15

**Authors:** Hira Saleem, Usman Ali Ashfaq, Habibullah Nadeem, Muhammad Zubair, Muhammad Hussnain Siddique, Ijaz Rasul

**Affiliations:** Department of Bioinformatics and Biotechnology, Government College University Faisalabad, Faisalabad, Pakistan; Bahauddin Zakariya University, PAKISTAN

## Abstract

*Stenotrophomonas maltophilia* is a multidrug resistant pathogen associated with high mortality and morbidity in patients having compromised immunity. The efflux systems of *S*. *maltophilia* include SmeABC and SmeDEF proteins, which assist in acquisition of multiple-drug-resistance. In this study, proteome based mapping was utilized to find out the potential drug targets for *S*. *maltophilia* strain k279a. Various tools of computational biology were applied to remove the human-specific homologous and pathogen-specific paralogous sequences from the bacterial proteome. The CD-HIT analysis selected 4315 proteins from total proteome count of 4365 proteins. Geptop identified 407 essential proteins, while the BlastP revealed approximately 85 non-homologous proteins in the human genome. Moreover, metabolic pathway and subcellular location analysis were performed for essential bacterial genes, to describe their role in various cellular processes. Only two essential proteins (Acyl-[acyl-carrier-protein]—UDP-N acetyl glucosamine O-acyltransferase and D-alanine-D-alanine ligase) as candidate for potent targets were found in proteome of the pathogen, in order to design new drugs. An online tool, Swiss model was employed to model the 3D structures of both target proteins. A library of 5000 phytochemicals was docked against those proteins through the molecular operating environment (MOE). That resulted in to eight inhibitors for both proteins i.e. enterodiol, aloin, ononin and rhinacanthinF for the Acyl-[acyl-carrier-protein]—UDP-N acetyl glucosamine O-acyltransferase, and rhazin, alkannin beta, aloesin and ancistrocladine for the D-alanine-D-alanine ligase. Finally the ADMET was done through ADMETsar. This study supported the development of natural as well as cost-effective drugs against *S*. *maltophilia*. These inhibitors displayed the effective binding interactions and safe drug profiles. However, further *in vivo* and *in vitro* validation experiment might be performed to check their drug effectiveness, biocompatibility and their role as effective inhibitors.

## Introduction

*Stenotrophomonas maltophilia* is an intensive emergent gram-negative bacterium of the human-ecological origin worldwide. That characteristically impart resistance to different classes of antibiotics and hefty metals [1, 2]. It is responsible for wide scope infections in clinics and local area settings including infection of respiratory tract and septicemia which are pervasive in nature. Whereas infections of bone, joints, urinary tract as well as meningitis are less successive [3].

Owing to adaptive behavior of intrinsic resistance of *S*. *maltophilia* through horizontal gene transfer and mutation against anti-microbial utilized beforehand, the medicinal approach is getting popular nowadays [4]. The resistance emerges fundamentally by modifying the medication targets, bypassing molecules, efflux pumps, substance alteration, self-prescription, mutations or by phenotypic variation arising internally or externally by the host [5]. The efflux systems of *S*. *maltophilia* include SmeABC and SmeDEF proteins, which assist in acquisition of multiple-drug-resistance [4]. Furthermore, that offer an extreme tendency of fighting against drugs by extraordinary liability outcomes such as trimethoprim/sulfamethoxazole (TMP/SMX), fluoroquinolones and ceftazidime [6, 7]. Consequently it is of imperative significance to identify novel and potent therapeutic targets in *S*. *maltophilia* to cope with this multidrug-resistant pathogen successfully.

The enormous progress in computational biology and diversified applications of bioinformatics have gained importance in drug designing thereby reducing the cost and time needed for *in vivo* screening and testing [8, 9]. The bioinformatics has substantially shortened traditional lab trials through employment of approaches including identification of drug candidates, structure-based designing of drug molecule, screening of antiviral drugs, comparative investigations utilizing genome to recognize host specific targets etc. [10, 11]. Currently the subtractive genomic approach is being focused in order to examine the entire host and proteome of bacterium. This is to recognize the proteins with various therapeutic perspectives solely present in the pathogenic genome, by excluding the homologous proteins of the host [12]. Numerous investigations have already utilized this approach on the multiple pathogenic strains and detailed fruitful identification and acknowledgment of novel species-specific therapeutic targets [13, 14].

The current study involves applying subtractive proteomics approach on the whole proteome of *S*. *maltophilia*. Briefly, the proteins which are fundamental to pathogenic survival were prioritized via computational tools and databases. It was followed by eliminating host homology proteins. Merely pathogenic proteins were retained to minimize the accidental therapeutic blockage by the host and involved in the metabolism of host. These proteins were further subjected to prediction of their subcellular localization for recognizing membrane protein followed by the drug-ability analysis. That led to the identification of two virtually hit compounds including Acyl-[acyl-carrier-protein]—UDP-N acetyl glucosamine O-acyltransferase and D-alanine-D-alanine ligase as therapeutic targets in *S*. *maltophilia*. These proteins were then docked with phytochemicals, enterodiol, aloin, ononin and rhinacanthinF. That revealed sound molecular interaction and high docking score as well as binding affinity. The potent compounds were also evaluated for drug-likeness and toxicity assessment that may serve as the target for the further optimization of the compounds through experimental study.

## Methods

**Fig 1:**
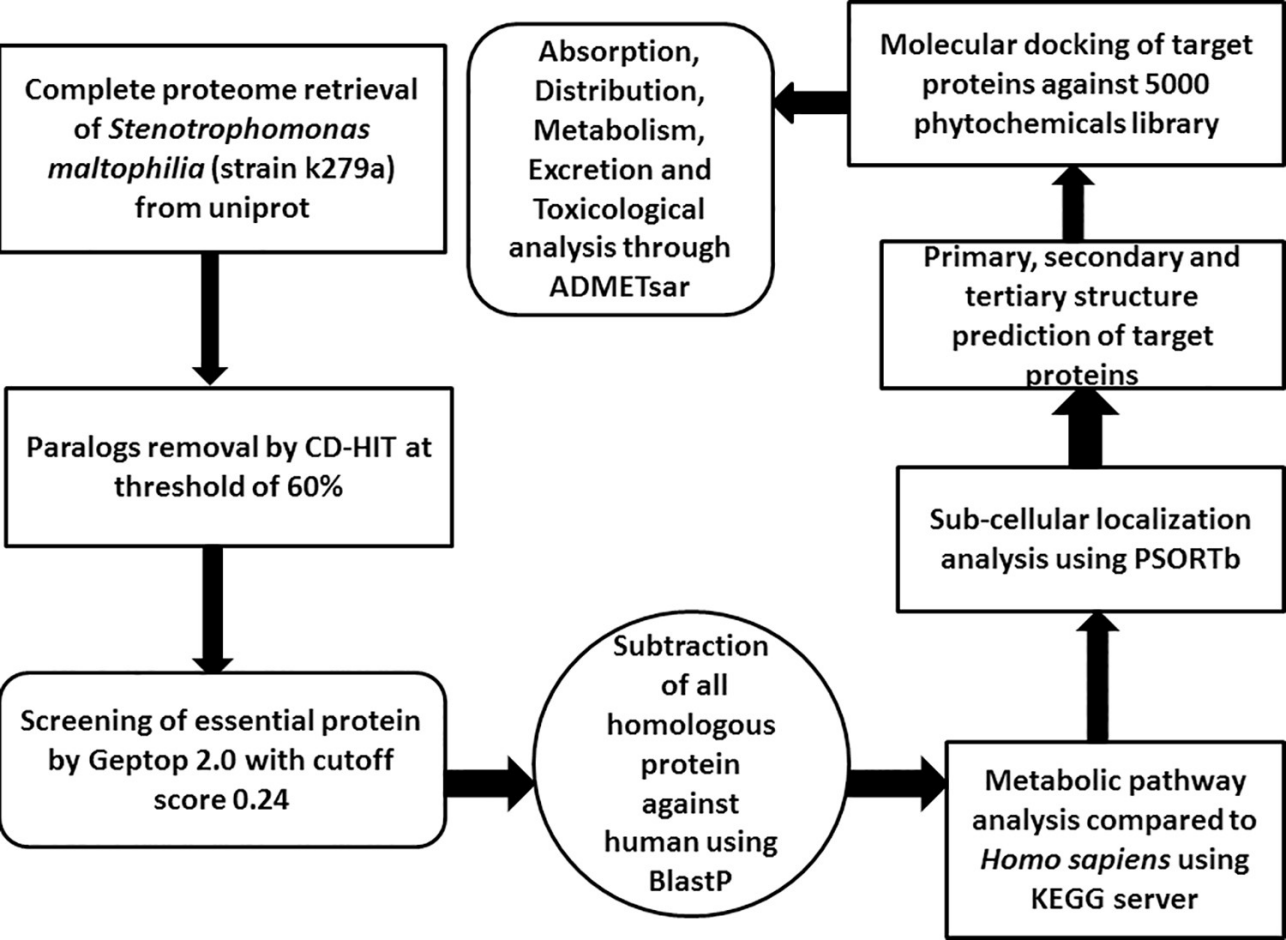
Overall flow chart of subtractive genomic against *S*. *maltophilia*. This shows analysis of whole proteome of *S*. *maltophilia* (strain k279a).

The subtractive genomic approach was employed for analysis of the whole proteome of *S*. *maltophilia* (strain k279a) screen immunogenic proteins that may serve as novel drug targets. The overall flowchart of the study is shown (Fig 1).

### The whole proteome retrieval

First of all the whole proteome of *S*. *maltophilia* (strain k279a) was retrieved from Uniprot in the FASTA format.

### Identification of paralogous sequences

The whole proteome of *S*. *maltophilia* (strain k279a) was subjected to CD-HIT suite. The parameters were set to default except for threshold value kept to 60%. CD-HIT suite is widely employed for comparing and clustering protein and genomic sequences. That is to remove paralogs or redundant proteins [15].

### Identification of essential proteins

The Geptop 2.0 server was used to retrieve essential proteins of *S*. *maltophilia*. That server is used for the detection of essential genes taking into account comparison of the phylogeny and orthology of provided query protein with datasets of essential genes.

### Identification of essential non-homologous proteins

The essential proteins were submitted to Blastpagainst host proteome with a threshold of e-value 10^−4^, with the query coverage and identity of more than 70% and 30%, respectively. The purpose was to identify those proteins which are non-homologous to the host.

### Analysis of metabolic pathways

The essential proteins of *S*. *maltophilia* were analyzed through KEGG automatic annotation server. The pathways unique to *S*. *maltophilia* (strain k279a) and absent in humans were selected [19] at KEGG [20].

### Subcellular localization analysis

The target proteins were subjected to the identification of subcellular localization of metabolic proteins of *S*. *maltophilia* by the PSORTb tool to enable identification of these predicted therapeutic targets.

### Selection of membrane proteins by drug-ability

In order to screen for the uniqueness of putative targets, Drug-Bank 5.1.0 database set to default limitations was used. Consequently, the proteins with significant hit higher to threshold with pre-treated drug targets displayed common functions. These were proceeded further for drug-able testing.

### Primary and secondary structure analysis of target proteins

The evaluations of primary structure of selected proteins were done through EXPASY. It was followed by prediction of secondary structure through PSIPRED. That generated outcome based on feed-forward neural networks [16].

Furthermore, SignalP-5.0 server was used for the prediction of location for signal peptide and their protein cleavage sites. Subsequently these targets were tested for transmembrane topology via TMHMM tool. Which relies on Hidden Markov model for the prediction and so on predicts transmembrane helices and precisely distinguishes soluble from membrane proteins [17].

### Structure prediction and validation

An online tool, Swiss-Model was employed to predict the 3D structure of putative proteins. That tool identifies the template, aligns it with the target sequence, constructs and evaluates quality of the 3D model [18]. The Chimera Structure Visualization software [19] was used to visualize and Galaxy WEB server was used to refine the models. The quality of model was evaluated using SAVES server which analyzes them on the basis of ERRAT [20], WHATCHECK [21] and PROCHECK [22].

### Compounds library preparation and molecular docking

For docking, the 2D conformation of compounds were downloaded from the PubChem [23] followed by protonation and energy minimization in MOE software and further added to the database. These compounds were then docked with putative proteins via the MOE software [24].

### Physiochemical property profiling and toxicity predictions

Molinspiration server was used to analyze the molecular descriptors and drug likeliness properties of compounds. In fact that gives a prediction based ‘rule of five’ (Ro5) [25]. AdmetSAR database was used to indicate the pharmacokinetic properties such as ADMET toxicity of the compounds [26]. ProTox-II webserver used for selected molecules were subjected to various toxicity screening endpoints models. That is a web server designed to predict the toxicity of various toxicological endpoints for different chemical compounds [27].

## Results

**Fig 2:**
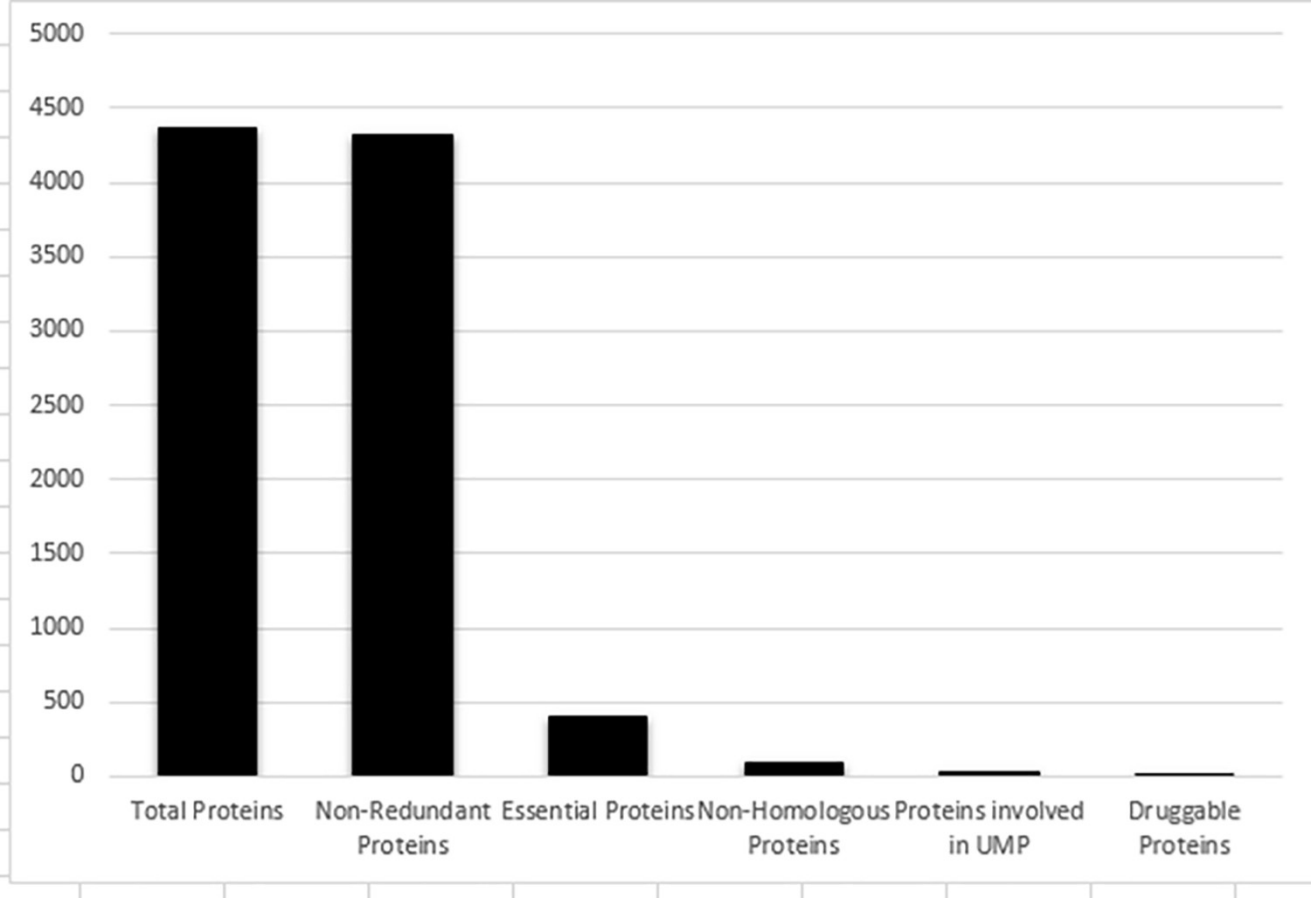
Summary for the detection of novel drug targets in *S*. *maltophilia*. This shows protein counts of selected paralogous sequences, essential proteins, non-homologous proteins and drug target proteins.

This study was done to recognize the novel drug targets in *S*. *maltophilia*. In this study, the subtractive genomic approach is employed for seeking therapeutic target proteins which are indispensable for the bacterial survival but absent in the host. The insight of that approach is shown (Fig 2).

### Selection of paralogous sequences

The proteome of *S*. *maltophilia* strain was based on 4365 proteins which were subsequently subjected to the CD-HIT server that facilitated selection of paralogous sequences. It was followed by excluding paralogous sequences showing similarity more than 60% thereby retaining back to 4315 non paralogous proteins.

### Selection of essential proteins

The screening of 4315 non-paralogous proteins using Geptop 2.0 server resulted in 407 essential proteins. Actually designing of antibacterial compounds relies on docking and hinders with essential proteins [28].

### Selection of non-homologous proteins

The cellular proteins of humans evolved to be homologous with bacteria [29] that necessitate the therapeutics to be non-homologous to humans to avoid cross-reactivity. Out of 407 proteins subjected to BlastP, 85 revealed as non-homologous.

### Metabolic pathway analysis

The analysis of those 85 non homologous proteins appeared to be involved in 33 pathways explored with KEGG. Among these 33 pathways, 13 were predicted to be particular for the *S*. *maltophilia* and remaining to be common for the *S*. *maltophilia* as well as host. Briefly, 27 essential proteins are revealed to participate in 13 pathways (Table 1). Among these 27 proteins which were analyzed in KEGG database, 24 proteins were found to participate in common metabolic pathways and rest of three namely chromosomal replication initiator protein DnaA, D-alanine-D-alanine ligase and Acyl-[acyl-carrier-protein]—UDP-N acetyl glucosamine O-acyltransferase were observed to participate in the pathogen specific pathways. Hence, continued further for analysis.

**Table 1:** Essential non-homologous proteins involved in 27 unique metabolic pathways.

SrNo.	Protein Name (ID)	Unique Pathway	Common pathway
1	Bifunctional protein (B2FHY5)	Sml00541-O-Antigen nucleotide sugar biosynthesis	Sml00520-Amino sugar and nucleotide sugar metabolism
			Sml01100-Metabolic pathways
2	Ubiquinone/menaquinone biosynthesis C-methyl transferase (B2FUU6)	sml01110-Biosynthesis of secondary metabolites	Sml01240 -Biosynthesis of cofactors
			Sml00130-Ubiquinone and other terpenoid-quinone biosynthesis
			sml01100 -Metabolic pathways
3	UDP-N-acetyl glucosamine 1-carboxyvinyltransferase (B2FRX1)	Sml00550-Peptidoglycan biosynthesis	sml01100-Metabolic pathways
			Sml00520-Amino sugar and nucleotide sugar metabolism
4	**D-alanine—D-alanine ligase (B2FNN9)**	Sml01502-Vancomycin resistance	
		Sml00550-Peptidoglycan biosynthesis	
		Sml00473-D-Alanine metabolism	
5	Phosphatidate cytidylyltransferase (B2FIA3)	sml01110-Biosynthesis of secondary metabolites	sml01100-Metabolic pathways
			sml00564-Glycerophospholipid metabolism
6	B2FNL3	sml01110-Biosynthesis of secondary metabolites	sml01100-Metabolic pathways
			sml01240- Biosynthesis of cofactors
			sml00740-Riboflavin metabolism
7	UDP-N-acetylmuramate—L-alanine ligase (B2FNN8)	Sml00550-Peptidoglycan biosynthesis	Sml00471-D-Glutamine and D-glutamate metabolism
			sml01100-Metabolic pathways
8	UDP-2,3-diacylglucosamine hydrolase (B2FQP4)	Sml00540- Lipopolysaccharide biosynthesis	sml01100-Metabolic pathways
9	**Acyl-[acyl-carrier-protein]—UDP-N acetyl glucosamine O-acyltransferase (B2FHN6)**	Sml01503- Cationic antimicrobial peptide (CAMP) resistance.	
		Sml00540- Lipopolysaccharide biosynthesis	
10	2-Dehydro-3-deoxyphosphooctonate aldolase (B2FK85)	Sml00540- Lipopolysaccharide biosynthesis	sml01100-Metabolic pathways
11	Histidine biosynthesis bifunctional protein (B2FPM1)	sml01110-Biosynthesis of secondary metabolites	sml01100-Metabolic pathways
			sml01230-Biosynthesis of amino acids
			sml00340-Histidine metabolism
12	Shikimate kinase (B2FQI7)	sml01110-Biosynthesis of secondary metabolites	sml01100-Metabolic pathways
			sml01230-Biosynthesis of amino acids
			sml00400-Phenylalanine, tyrosine and tryptophan biosynthesis
13	Acetyl-coenzyme A carboxylase (B2FHN1)	sml01110-Biosynthesis of secondary metabolites	Sml00061-Fatty acid biosynthesis
		sml01120-Microbial metabolism in diverse environments	sml01100-Metabolic pathways
			sml01200- Carbon metabolism
			sml01212-Fatty acid metabolism
			sml00640- Propanoate metabolism
			sml00620- Pyruvate metabolism
14	Glutamyl-tRNA reductase (B2FQ15)	sml01110-Biosynthesis of secondary metabolites	sml01100-Metabolic pathways
		sml01120-Microbial metabolism in diverse environments	Sml01240 -Biosynthesis of cofactors
			Sml00860- Porphyrin and chlorophyll metabolism
15	3-Methyl-2-oxobutanoate hydroxymethyltransferase (B2FL67)	sml01110-Biosynthesis of secondary metabolites	Sml00770- Pantothenate and CoA biosynthesis
			sml01100-Metabolic pathways
			Sml01240 -Biosynthesis of cofactors
16	4-Hydroxy-3-methylbut-2-enyl diphosphate reductase (B2FU83)	sml01110-Biosynthesis of secondary metabolites	Sml00900- Terpenoid backbone biosynthesis
			sml01100-Metabolic pathways
17	Chorismate synthase (B2FP01)	sml01110-Biosynthesis of secondary metabolites	sml01100-Metabolic pathways
			sml01230-Biosynthesis of amino acids
			sml00400-Phenylalanine, tyrosine and tryptophan biosynthesis
18	**Chromosomal replication initiator protein DnaA (B2FUW1)**	Sml02020- Two-component system	
19	3-deoxy-manno-octulosonate cytidylyltransferase (B2FK23)	Sml00540- Lipopolysaccharide biosynthesis	sml01100-Metabolic pathways
20	Tetraacyldisaccharide 4’-kinase (B2FK22)	Sml00540- Lipopolysaccharide biosynthesis	sml01100-Metabolic pathways
21	Oxygen-dependent coproporphyrinogen-III oxidase (B2FND0)	sml01110-Biosynthesis of secondary metabolites	sml01100-Metabolic pathways
			sml00860-Porphyrin and chlorophyll metabolism
			Sml01240 -Biosynthesis of cofactors
22	Pantothenate synthetase (B2FL68)	sml01110-Biosynthesis of secondary metabolites	sml01100-Metabolic pathways
			sml00770-Pantothenate and CoA biosynthesis
			Sml01240 -Biosynthesis of cofactors
			Sml00410- beta-Alanine metabolism
23	Protein translocase subunit SecA (B2FPB2)	Sml02024- Quorum sensing	Sml03060- Protein export
		Sml03070- Bacterial secretion system	
24	Acetyl-coenzyme A carboxylase carboxyl transferase subunit beta (B2FNY8)	sml01110-Biosynthesis of secondary metabolites	sml01100-Metabolic pathways
		sml01120-Microbial metabolism in diverse environments	sml01212-Fatty acid metabolism
			sml00640- Propanoate metabolism
			sml00620- Pyruvate metabolism
			Sml00061-Fatty acid biosynthesis
			sml01200- Carbon metabolism
25	Succinyl-diaminopimelate desuccinylase (B2FIC0)	Sml00300- Lysine biosynthesis	sml01100-Metabolic pathways
		sml01120-Microbial metabolism in diverse environments	sml01230-Biosynthesis of amino acids
26	4-hydroxy-tetrahydrodipicolinate reductase (B2FQ70)	Sml00300- Lysine biosynthesis	sml01100-Metabolic pathways
		sml01120-Microbial metabolism in diverse environments	sml01230-Biosynthesis of amino acids
		sml01110-Biosynthesis of secondary metabolites	
		sml00261- Monobactam biosynthesis	
27	Glycerol-3-phosphate dehydrogenase [NAD(P)+] (B2FHD8)	sml01110-Biosynthesis of secondary metabolites	Sml00564-Glycerophospholipid metabolism

### Subcellular localization prediction

The prediction of subcellular location is a quick way to obtain protein as it facilitates the steps required to purify in the experimental setup. That is done by determining its location i.e. whether cytoplasmic or membranous. The prediction retrieved via PSORTb revealed those proteins to be cytoplasmic in nature (Table 2).

**Table 2:** Sub cellular localization prediction of proteins involved in unique metabolic pathways.

Protein ID (Protein Name)	Localization prediction	Drug-able
B2FNN9 (D-alanine—D-alanine ligase)	Cytoplasmic	Yes
B2FHN6 **(**Acyl-[acyl-carrier-protein]—UDP-N acetyl glucosamine O-acyltransferase)	Cytoplasmic	Yes
B2FUW1 (Chromosomal replication initiator protein DnaA	Cytoplasmic	Yes

### Selection of drug-able proteins

Those 3 putative proteins were subjected to the Drug Bank. Two of them were found to be significantly similar to drug entries of the database, either to FDA approved or experimental drugs. These might act as potential novel drug targets (Table 3).

**Table 3:** Drug-able target proteins analysed via DrugBank.

Sr. No.	Accession No.	Protein Name	DrugBank ID	Drug Name	Category	Organism
1	B2FNN9	D-alanine—D-alanine ligase	DB07805	3-CHLORO-2,2-DIMETHYL-N-[4 (TRIFLUOROMETHYL)PHENYL]PROPANAMIDE	Experimental Approved	*Staphylococcus aureus*(strainCOL)
			DB00260			
						*Escherichia coli* (strain K12)
				Cycloserine		
2	B2FHN6	Acyl-[acyl-carrier-protein]—UDP-N acetyl glucosamine O-acyltransferase	DB01694	D-tartaric acid	Experimental	*Helicobacter-pylori* (strain ATCC 700392 /26695)
			DB08558	2-HYDROXYMETHYL**-6-**OCTYLSULFANYL-TETRAHYDRO-PYRAN-3,4,5-TRIOL	Experimental	
						*Helicobacter pylori* (strain ATCC 700392 / 26695)

### Structural analysis of target protein

The analysis of primary structure revealed that the D-alanine—D-alanine ligase protein and Acyl-[acyl-carrier-protein]—UDP-N acetyl glucosamine O-acyltransferase harbor the molecular mass of 21.07 kDaand 28.1 kDa, respectively. Moreover, their isoelectric points were 4.87 and 6.47, grand averages of hydropathic (GRAVY) were 0.004 and -0.100, terminating amino acid at the N-terminus of protein was lysine and methionine and the instability indices were 30.98 and 18.93, respectively. As these proteins carried isoelectric points below 7 so that is an indication of positively charged proteins. Moreover, the GRAVY computed values explained them as hydrophilic and unstable.

Further, the secondary structure of Acyl-[acyl-carrier-protein]—UDP-N acetyl glucosamine O-acyltransferase and D-alanine-D-alanine ligase protein showed that they have 27.38% and 32.19% of alpha helices, 24.33% and 18.44% extended, and 48.29% and 49.38% of random coils, respectively. Both proteins displayed no beta turns.

The signal peptide probability values of Acyl-[acyl-carrier-protein]—UDP-N acetyl glucosamine O-acyltransferase and D-alanine-D-alanine ligase were obtained by SignalP. These values were 0.012 and 0.021, respectively. That was indicating the absence of signal peptide in these protein targets. The TMHMM showed absence of transmembrane helices in both putative proteins.

### The 3D structure prediction and validation

**Fig 3:**
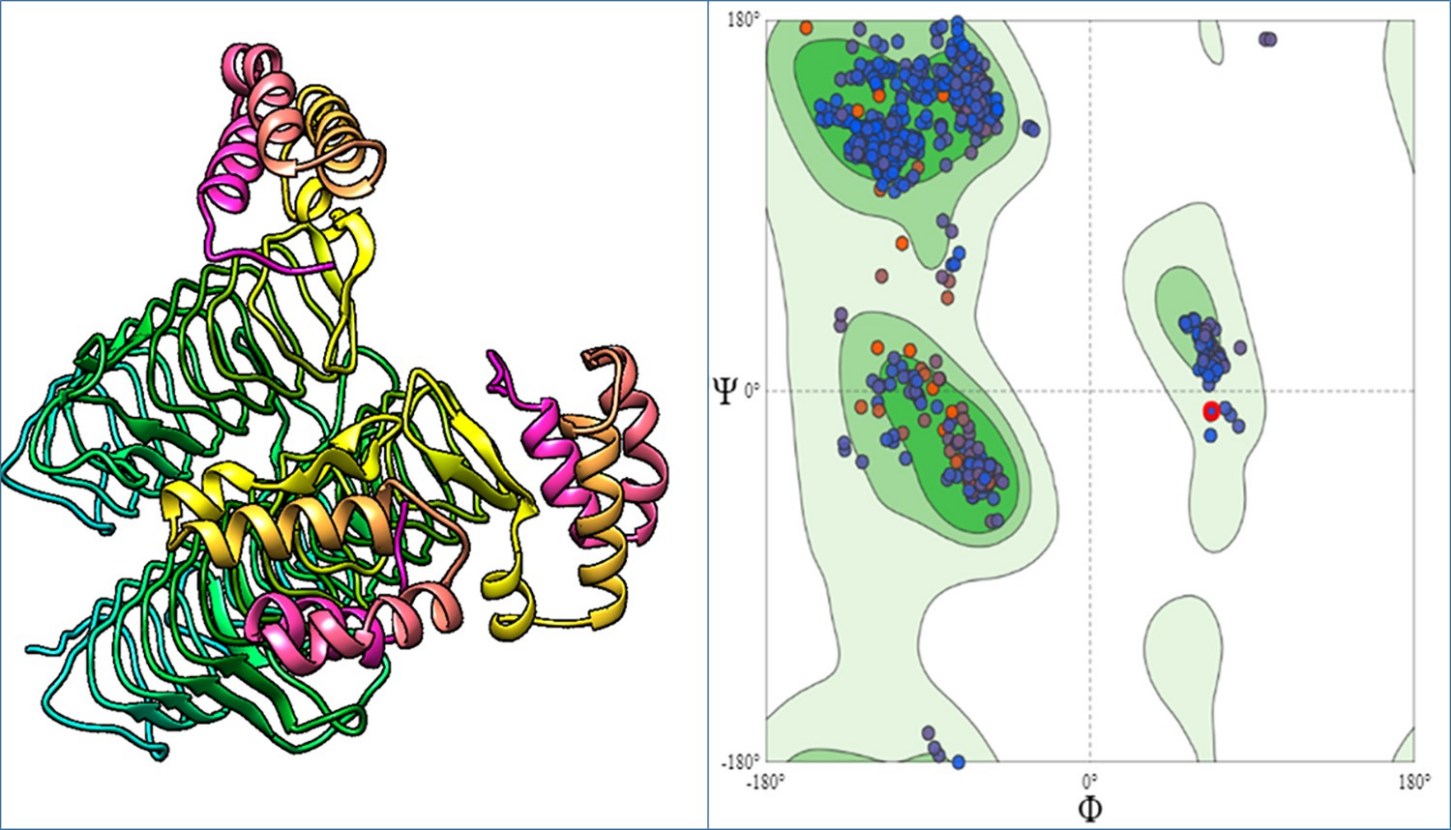
Structure of the D-alanine-D-alanine ligase protein. (A) Three dimensional structure of D-alanine-D-alanine ligase protein. (B) Ramachandran Plot of D-alanine-D-alanine ligase protein.

**Fig 4:**
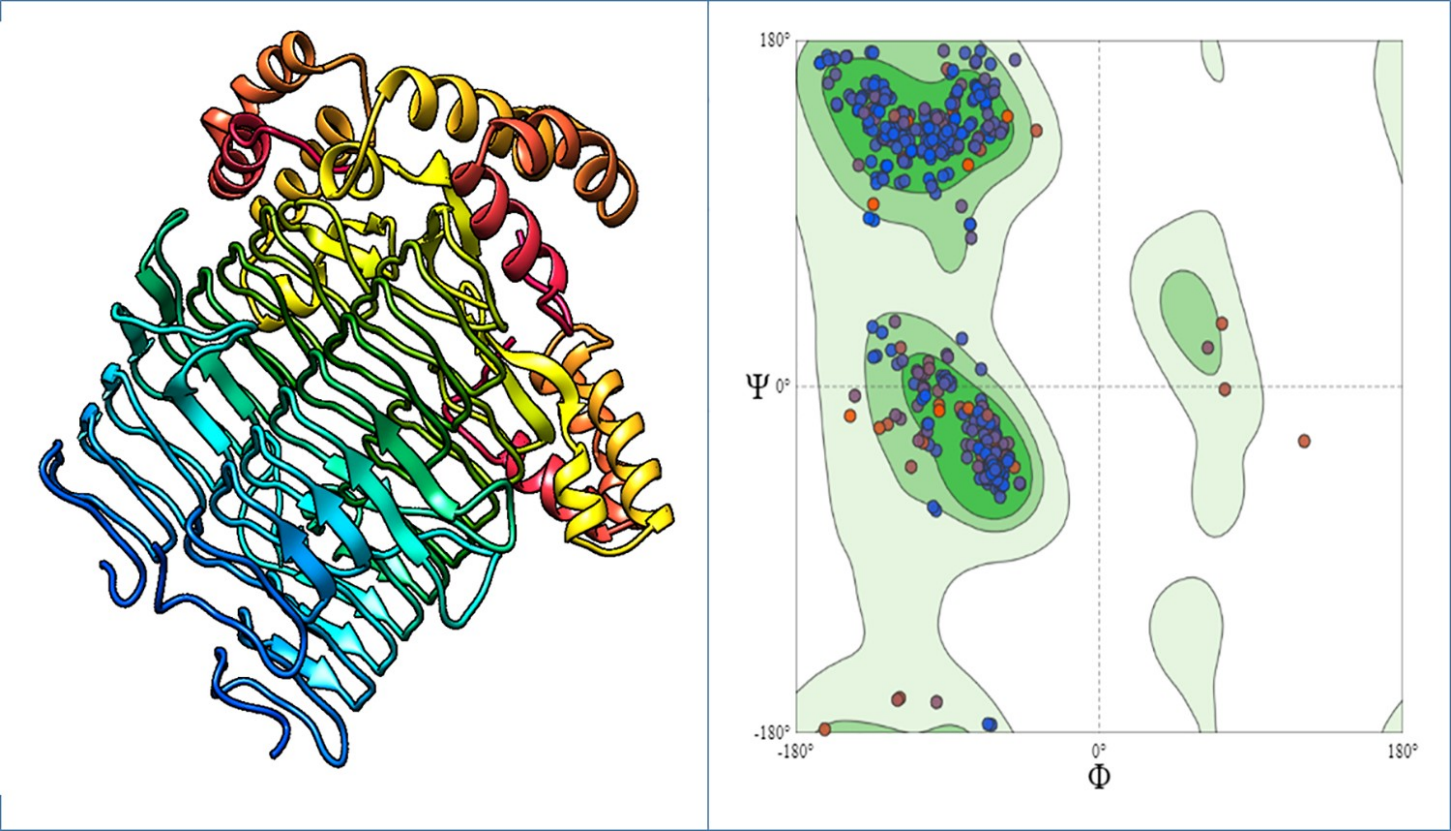
Structure of the Acyl-[acyl-carrier-protein]—UDP-N acetyl glucosamine O-acyltransferase protein. (A) Three dimensional structure of Acyl-[acyl-carrier-protein]—UDP-N acetyl glucosamine O-acyltransferase protein. (B) Ramachandran Plot of Acyl-[acyl-carrier-protein]—UDP-N acetyl glucosamine O-acyltransferase protein.

The structures of both proteins Acyl-[acyl-carrier-protein]—UDP-N acetyl glucosamine O-acyltransferase and D-alanine-D-alanine ligase were predicted by the Swiss-Model with confidence level of 100% and coverages were 92% and 98%, respectively. The SAVES analyzed those models by visualizing through Verify 3D, WHATCHECK, Prove, PROCHECK and ERRAT. The RAMPAGE server generated the Ramachandran plot for Acyl-[acyl-carrier-protein]—UDP-N acetyl glucosamine O-acyltransferase. This displayed that 96.9% of residues were found in the most favored regions, 0.3% of residues in additional allowed regions, 2.8% of residues in generously allowed region and none in disallowed region. According to the Ramachandran plot generated for D-alanine-D-alanine ligase, 85.8% of residues were found in the most favored region, while 13.7% and 13.2% of amino acids resided in additional allowed regions, and 0.8% and 1.0% of residues are found in generously allowed region and none in disallowed region (Figs 3 & 4).

### Molecular docking

**Fig 5:**
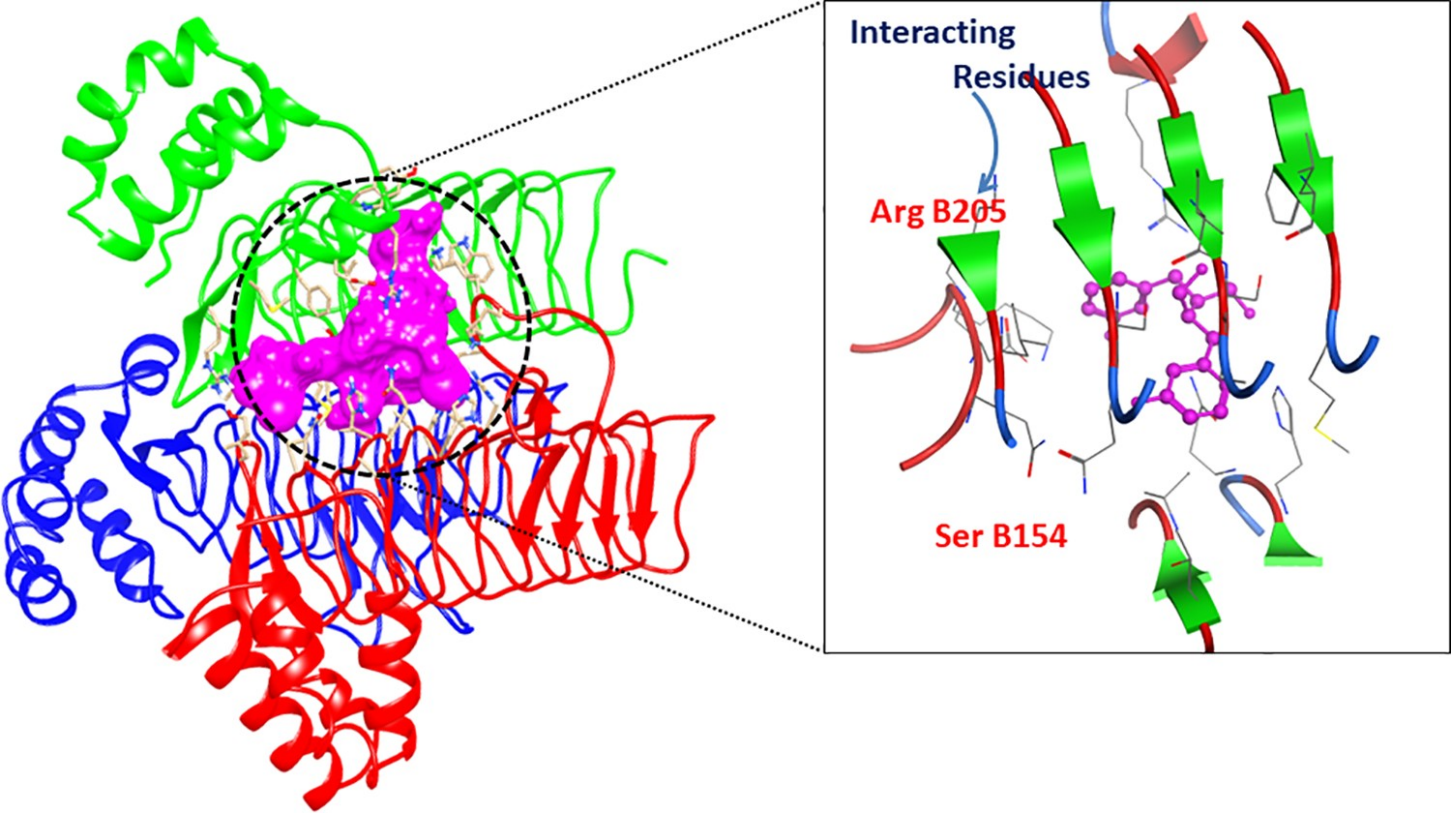
2D and 3D interaction diagram of Acyl-[acyl-carrier-protein]—UDP-N acetyl glucosamine O-acyltransferase protein. The structure shows complex with Enterodiol.

**Fig 6:**
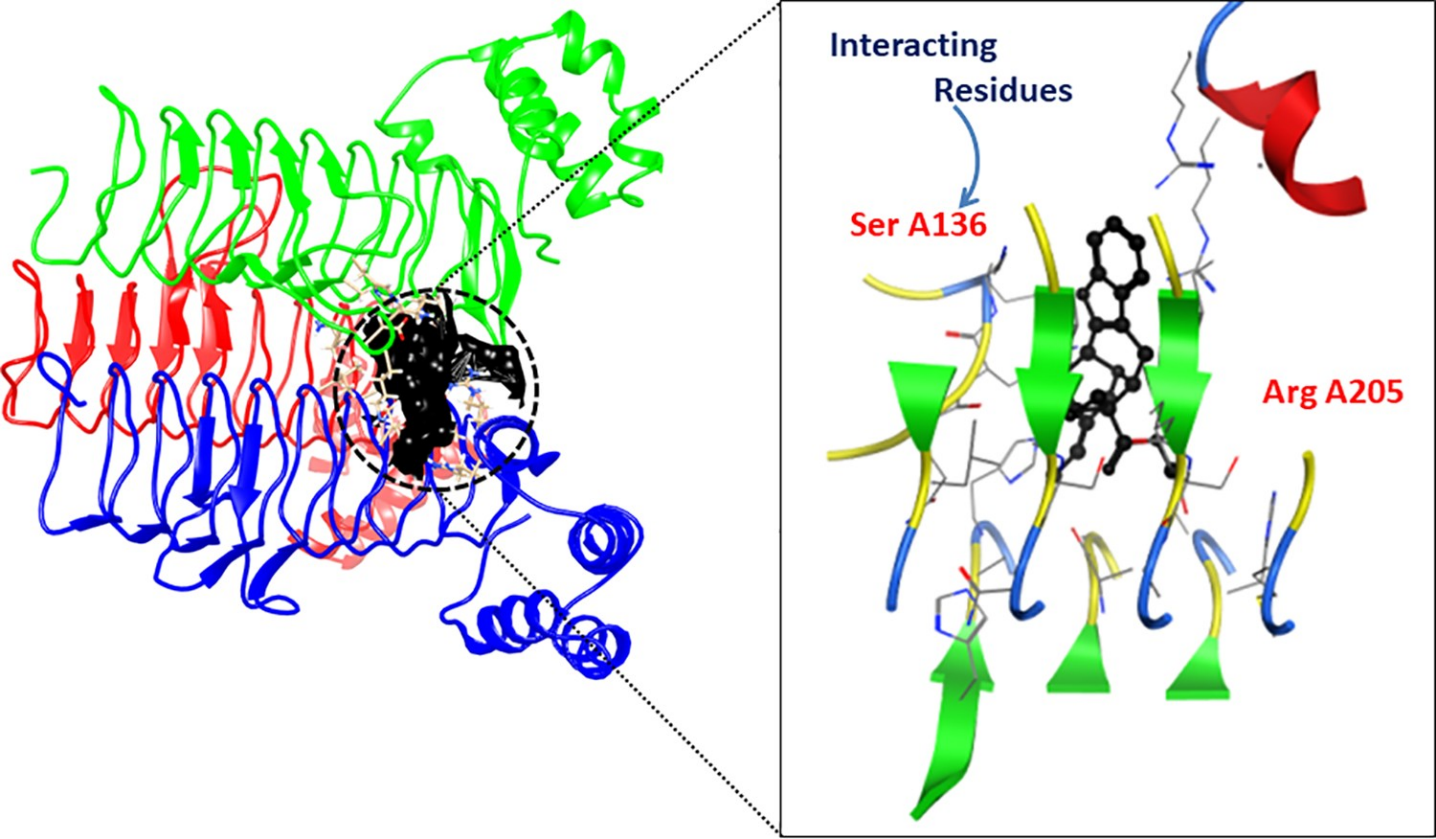
2D and 3D interaction diagram of D-alanine-D-alanine ligase protein. The structure shows complex with Rhazin.

The minimum binding energy and scoring function of each docked ligand are shown (Table 4). The LigX interaction diagrams showed that Acyl-[acyl-carrier-protein]—UDP-N acetyl glucosamine O-acyltransferase (B2FHN6) interacts with the enterodiol, aloin, ononin and rhinacanthinF having binding scores -11.36, -17.44, -15.42 and -12.86, respectively. While D-alanine-D-alanine ligase (B2FNN9) interacted with ligands including rhazin, alkannin beta, aloesin and ancistrocladine having the docking scores -12.58, -12.57, -14.39 and -14.41, respectively. All these compounds exhibited RMSD below 3 which is an indication of the sound interaction. The 2D and 3D interaction diagrams are displayed (Figs 5 & 6).

**Table 4:** The table displays the docking score and RMSD value for the compounds.

PubChem ID	Compound Name	S-score	RMSD_Refine
115089	Enterodiol	-11.36	3.0
14989	Aloin	-17.44	1.7
442813	Ononin	-15.42	2.5
10411189	RhinacanthinF	-12.86	1.4
21160714	Rhazin	-12.58	2.4
442720	Alkannin beta, beta-dimethylacrylate	-12.57	1.4
160190	Aloesin	-14.39	2.6

### ADMET/Drug scans results

The drug likeliness of compounds was predicted through the Molinspiration server, based on the Ro5. The selected candidates indicate zero violations to Lipinski’s Ro5 and showed acceptable drug-like properties (Table 5). All candidate compounds were assessed for the pharmacokinetic properties through the AdmetSAR server for drug likeliness (Table 6).

**Table 5:** Results of inhibitors examined for Lipinski rule.

PubChem ID	Compound Name	Molecular Weight	Number of HBA	Number of HBD	MlogP
115089	Enterodiol	302.37	4	4	2.10
14989	Aloin	418.40	7	3	0.89
442813	Ononin	430	9	4	0.65
10411189	RhinacanthinF	444.44	9	0	2.52
21160714	Rhazin	352.43	4	2	2.57
442720	Alkannin beta, beta-dimethylacrylate	370.40	6	2	3.67
160190	Aloesin	394.38	9	5	-0.55
161741	Ancistrocladine	407.51	5	2	5.14

**Table 6:** ADMET properties for compounds as predicted by admetSAR server.

Compounds	Enterodiol	Aloin	Ononin	RhinacanthinF	Rhazin	Alkannin beta, beta-dimethylacrylate	Aloesin	Ancistrocladine
**Absorption**								
Blood Brain Barrier	**+**	**-**	**-**	**-**	**+**	**-**	**-**	**-**
Human Intestinal Absorption	**+**	**+**	**-**	**+**	**+**	**+**	**+**	**+**
**Metabolism**								
P-glycoprotein substrate	**-**	**-**	**-**	**-**	**+**	**-**	**-**	**+**
CYP1A2 Inhibitor	No	No	No	Yes	Yes	Yes	No	No
CYP 450 2C9 Inhibitor	No	No	No	Yes	No	Yes	No	No
CYP 450 2D9 Inhibitor	No	No	No	No	No	No	No	Yes
CYP 450 2C19 Inhibitor	Yes	No	No	Yes	No	Yes	No	No
CYP 450 3A4 Inhibitor	No	No	No	Yes	No	No	Yes	Yes
**Distribution**								
**Subcellular Localization**	Mitocho-ndria	Mitocho-ndria	Mitochon-dria	Mitochon-dria	Mitochon-dria	Mitochon-dria	Mitochon-dria	Mitochon-dria
**Toxicity**								
**AMES Toxicity**	No	No	No	No	No	No	No	No

### Toxicity assessment

The rat oral acute toxicity (LD50) as mg/kg, toxicity classes (I–VI) predicted with accuracy in percent, the prediction of hepatotoxicity and cytotoxicity with their probability are indicated (Table 7).

**Table 7:** Prediction of class and accuracy, organ toxicity, oral acute toxicity and genetic toxicity endpoints of candidate compounds.

Sr. No	Compound name	Oral LD50 value (mg/Kg)	Predicted toxicity class	Prediction accuracy (%)	Hepato-toxicity	Proba-bility	Cytotoxicity	Proba-bility
1	Enterodiol	2950	V	69.26%	Inactive	0.80	Inactive	0.93
2	Aloin	221	III	68.07%	Inactive	0.85	Inactive	0.83
3	Ononin	3100	V	64.71%	Inactive	0.83	Inactive	0.58
4	RhinacanthinF	4000	V	68.07%	inactive	0.83	Inactive	0.95
5	Rhazin	300	III	68.07%	Inactive	0.85	Inactive	0.69
6	Alkannin beta, beta-dimethylacrylate	1000	IV	72.9%	Inactive	0.54	Inactive	0.88
7	Aloesin	832	IV	67.38%	Inactive	0.80	Inactive	0.78
8	Ancistrocladine	450	IV	69.26%	inactive	0.64	Inactive	0.51

**Fig 7:**
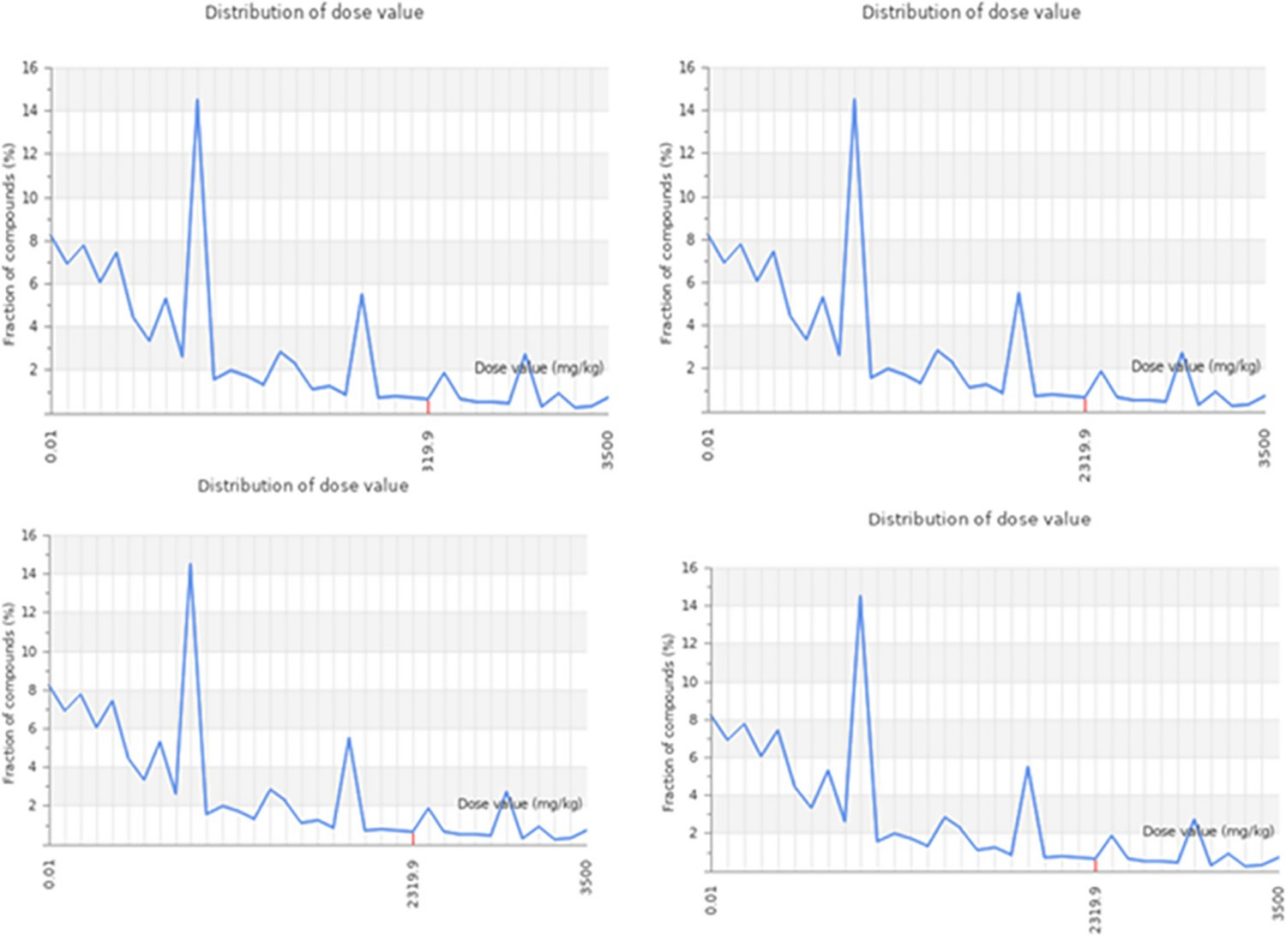
Graphical representation of predicted dose value distribution for Acyl-[acyl-carrier-protein]—UDP-N acetyl glucosamine O-acyltransferase protein. In this graph, x-axis represents distribution of dose value and y-axis represents fraction of compounds.

**Fig 8:**
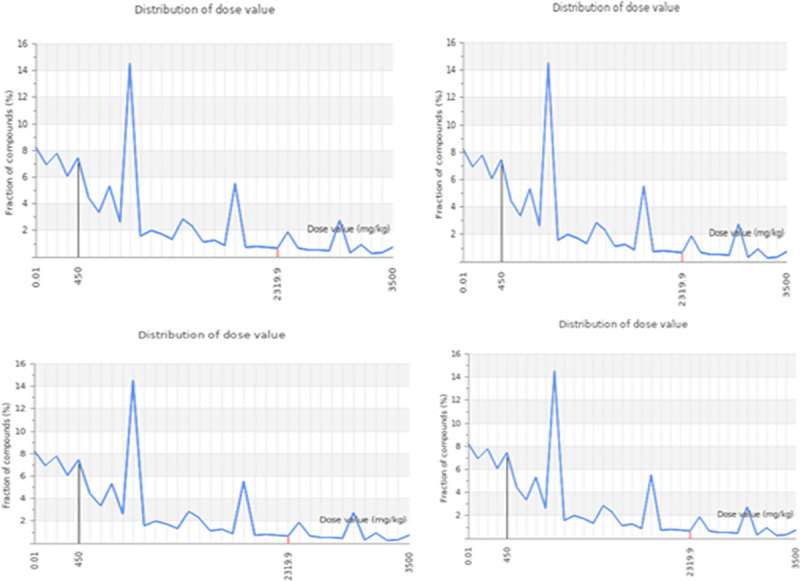
Graphical representation of predicted dose value distribution for D-alanine-D-alanine ligase. In this graph, x-axis represents distribution of dose value and y-axis represents fraction of compounds.

Among these compounds, enterodiol, ononin and rhinacanthinF exhibited the highest toxicity. Those belong to the class V i.e. prescribed as harmful when swallowed (2000 < LD50 ≤ 5000) with accuracy of 69.26%, 64.71% and 68.7%, respectively. While ononin predicted to have class III (50 < LD50 ≤ 300) prescribed as toxic if swallowed with accuracy of 68.07%. Moreover, other compounds like alkannin beta, aloesin and ancistrocladine belonging to class IV i.e prescribed as harmful after swallowing (300 < LD50 ≤ 2000) with accuracy of 72.9%, 67.38% and 69.26%, respectively. While the compound Rhazin was predicted to have class III (50 < LD50 ≤ 300) prescribed as toxic if swallowed with accuracy of 68.07%. All these compounds were predicted to show hepatotoxicity and cytotoxicity inactive with probability values are shown (Figs 7 & 8).

## Discussion

*Stenotrophomonas maltophilia* (strain k279a) is a multidrug-resistant (MDR) bacterium. There is currently no effective vaccine for that but frequent and thorough hand washing can prevent person-to-person transmission [1]. Recent advances in the disciplines of bioinformatics as well as computational biology have created a variety of approaches to drug designing and *in silico* analysis, reducing the time and expenses associated with trial and error of ions devoted to drug development [30].

The whole proteome of *S*. *maltophilia* (strain k279a) contained 4365 proteins, was analyzed through CD-HIT that eliminated all the redundant proteins and provided a group of 4315 non-redundant proteins. For the survival of bacteria, essential genes are necessary [31]. Essential genes are preferred targets for vaccine development and antibacterial drugs [32]. Thus 407 essential genes were screened from non-redundant proteins. These genes could be homologous to human [33]. Thus, targeting such genes may interfere with human metabolism and might be fatal. The possibility of cross-reactivity as well as adverse events might be reduced by the selection of non-homologous proteins that are not found in *Homo sapiens* [34]. To avoid such undesirable circumstances and toxicity, we screened 85 non-homologous proteins. It might be the best strategy to target and develop inhibitors against non-homologous sequences for the production of new drugs [35].

Only two proteins Acyl-[acyl-carrier-protein]—UDP-N acetyl glucosamine O-acyltransferase and D-alanine-D-alanine ligase were involved in a unique metabolic pathway. Different tools were applied to determine the sequence and structural features as well as functions and localization of that protein. Both proteins were found to be cytoplasmic as predicted by PSORTb [36]. A proper identification of the potential drug targets and inhibitors is crucial for the treatment of this disease due to their emerging multidrug resistance (MDR) patterns. In this study, a systematic subtractive approach was implemented for the identification of novel therapeutic targets of *S*. *maltophilia* through genome-wide metabolic pathway analysis of the essential genes and proteins. ADMET analyses were also made for the identification of potential inhibitors as well. Then, we found unique proteins as novel targets. Therapeutic targets and its inhibitors might give some breakthrough to treat *Stenotrophomonas maltophilia* efficiently in in vitro [37].

An online tool, Swiss-model was employed to model the 3D structure of Acyl-[acyl-carrier-protein]—UDP-N acetyl glucosamine O-acyltransferase and D-alanine-D-alanine ligase proteins [38]. The prediction of 3D structures provided the great aid in studying protein functions, dynamics, ligand interactions and other protein components [39]. Analysis of the Ramachandran plot showed that most residues were present in the acceptable as well as favored areas and few residues in the disallowed regions [40]. The ERRAT quality factor and z-score proved that structures of the Acyl-[acyl-carrier-protein]—UDP-N acetyl glucosamine O-acyltransferase and D-alanine-D-alanine ligase protein were of good quality.

Molecular docking was performed to find out the compounds exhibiting the best residue interaction with the target protein [26]. Out of 5000 docked molecules, eight (8) top molecules for both proteins: enterodiol, aloin, ononin, rhinacanthinF, rhazin, alkannin beta, aloesin and ancistrocladine were selected based on low score i.e. rmsd < 3 and different interacting residues. Based on "Lipinski’s Rule of Five" molecular profile and drug probability of these eight compounds were assessed. Those compounds were then tested for penetration of the blood-brain barrier (BBB), Human intestinal absorption (HIA) as well as AMES monitoring. Predicting the ADMET properties is a significant indicator of the behavior, toxicity level and fate of the drug candidate in the human body [41]. It provides a likelihood of the candidate’s ability to enter the intestinal absorption, metabolism, blood-brain barrier, subcellular localization and most significantly the level of harm that it can cause to the body [42]. The superfamily cytochrome P450 consists of isoforms such as CYP2A6, CYP1A2, CYP2C9, CYP2D6, CYP2C19, CYP3A4 and CYP2E1 which are involved in drug metabolism as well as hepatic clearance. So, inhibiting the cytochrome P450 isoforms can result in drug-drug interaction that hinders the metabolism of concomitant drugs that cause its accumulation to toxic levels [43]. Admet SAR showed that drugs exhibit localization in mitochondria. The compound localized in mitochondria show no toxicity. The ADMET profile of those compounds indicated that they have no adverse effects on absorption [44].

Various toxicity modules were subjected to the eight compounds obtained after the virtual screening [42]. Toxicity evaluation results revealed that none of the compounds was found to be cytotoxic, hepatotoxic as well as mutagenic [43].

## Conclusions

The subtractive genomics approach in our study has indicated two proteins of *S*. *maltophilia* as novel drug targets. The probability of cross reactivity seem to be ruled out between drugs and host proteins because there was no similarity between the proteome and ‘anti-targets’. Thus development of the putative target against *S*. *maltophilia* might be significantly effective for the eradication of otherwise resulting disease.
